# Perioperative Management of Subarachnoid Hemorrhage in a Patient with Alagille Syndrome and Unrepaired Tetralogy of Fallot: Case Report

**DOI:** 10.3389/fsurg.2017.00072

**Published:** 2017-12-04

**Authors:** Juan Fiorda-Diaz, Muhammad Shabsigh, Galina Dimitrova, Suren Soghomonyan, Gurneet Sandhu

**Affiliations:** ^1^Department of Anesthesiology, The Ohio State University Wexner Medical Center, Columbus, OH, United States

**Keywords:** Alagille syndrome, unrepaired Tetralogy of Fallot, subarachnoid hemorrhage, ruptured aneurysm, general anesthesia

## Abstract

Alagille syndrome (ALGS) is a genetic disorder associated with multisystem dysfunction involving the hepatic, cardiovascular, and neurologic systems. Tetralogy of Fallot (TOF), a congenital cardiac anomaly, is commonly found in these patients. Patients with ALGS may also have an increased risk of cerebrovascular abnormalities and bleeding. Ruptured cerebral aneurysm and subarachnoid hemorrhage (SAH) may be developed, increasing the incidence of morbidity and mortality. Advances in neuroimaging and neurosurgery have allowed early identification and treatment of such vascular abnormalities, improving patients’ outcomes and reducing life-threatening complications such as intracranial bleeding. Authors describe the perioperative management of a patient with ALGS and TOF who was admitted to the emergency department due a ruptured intracranial aneurysm with concomitant SAH. Surgical treatment included diagnostic cerebral arteriography with coil embolization of a left posterior communicating artery aneurysm, and placement of right external ventricular drain (EVD). The combination of neuroprotective anesthetic techniques, fast emergence from anesthesia, and maintenance of intraoperative hemodynamic stability led to a successful perioperative management. A multidisciplinary approach in specialized centers is essential for the treatment of patients with SAH, especially in patients with ALGS and complex congenital heart disease such as TOF.

## Introduction

Alagille syndrome (ALGS), Alagille–Watson syndrome, or “arterio-hepatic dysplasia” is an autosomal-dominant condition characterized by neonatal jaundice and abnormal intrahepatic bile ducts on liver histology caused by defects in the Notch signaling pathway (1). ALGS is commonly associated with an impaired hepatic, cardiac, renal, and central nervous system function (1). Diagnostic criteria were first established by Alagille et al. (2) followed by Watson and Miller (3).

Jagged 1 mutation was found in 94% of phenotypic patients with ALGS (4). However, clinical criteria remain the diagnostic hallmark of this disorder. Tetralogy of Fallot (TOF)—characterized by pulmonary artery stenosis (PAS), right ventricular hypertrophy (RVH), ventricular septal defect (VSD), and overriding aorta—is a concomitant clinical finding in about 12% of patients with ALGS (5).

Intracranial bleeding has been identified in 14% of patients with ALGS either by imaging or in autopsies. Furthermore, cerebrovascular accident and subarachnoid hemorrhage (SAH) are the most common intracranial bleeding events occurring in patients with ALGS (6). Complex congenital heart conditions (e.g., TOF and pulmonary atresia) are associated with an increased mortality in patients with ALGS as compared with non-ALGS patients (6).

Authors describe the perioperative management of a ruptured intracranial aneurysm with concomitant SAH in a patient with ALGS and unrepaired TOF. Risks and benefits of current surgical and anesthetic techniques should be cautiously identified in order to determine perioperative goals in this patient setting (7).

## Case Summary

We present a case of a 17-year-old female patient with SAH, ALGS, and unrepaired TOF. Past surgical history included open right ventricular outflow tract (RVOT) patch augmentation, pulmonary artery stenting, and craniotomy due to intracranial epidural hematoma.

The patient arrived to the emergency department (ED) complaining about the “worst headache of her life” and significant back and neck pain starting the day before. She was awake and able to provide some medical history despite being drowsy. She denied any focal numbness, weakness, loss of vision, or other neurologic deficits (Hunt and Hess grade II) (8). Significant findings on physical examination were as follows: ill appearing, slurred speech, and low weight (36.4 kg). Blood pressure (BP) and heart rate (HR) were normal whereas oxygen saturation (SpO_2_) was 92% (receiving complementary oxygen through a nasal cannula 2 L/min).

**Figure 1:**
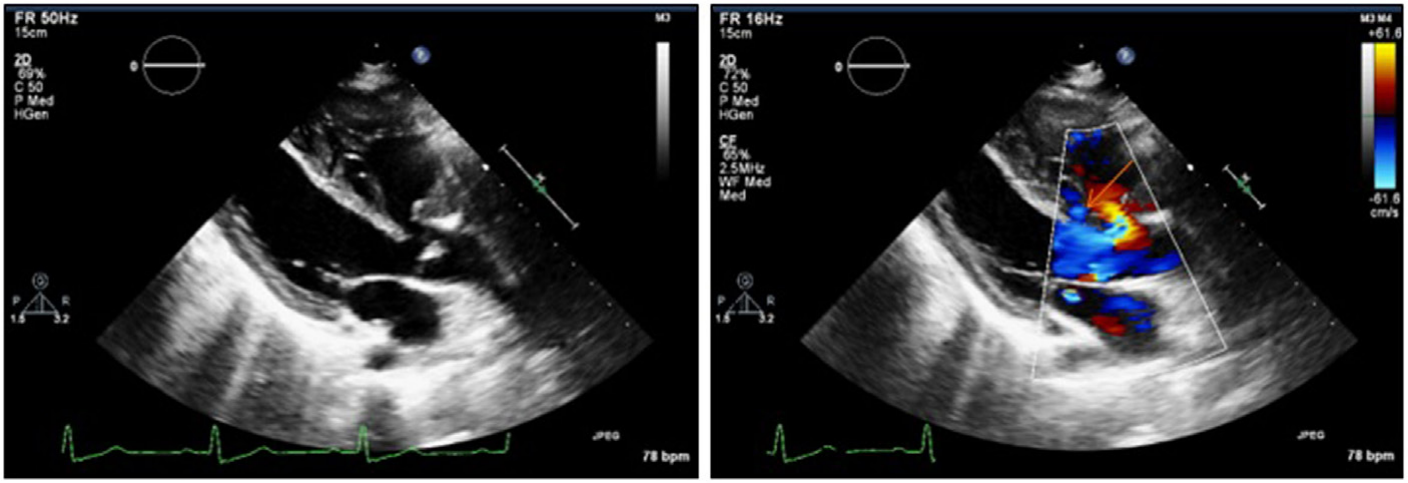
Preoperative transthoracic echocardiogram showing a membranous ventricular septal defect with predominant right to left shunting (see orange arrow).

Laboratory results were unremarkable. The electrocardiogram showed sinus rhythm, right-axis deviation, and RVH. The chest X-ray revealed mild-sub-segmental atelectasis in the right base, postoperative changes with sternotomy wires, and stent overlying the left hilum; heart size was toward the upper normal limit. A transthoracic echocardiogram showed a decreased left ventricular chamber size with hyperdynamic systolic function, left ventricular ejection fraction >65%, enlarged right ventricle with interventricular septal flattening (consistent with right ventricular overload), VSD with predominant right to left shunting and tricuspid regurgitation, and an estimated right ventricular systolic pressure of 107 mmHg (based on right atrial pressure of 15) (Figure 1). Severe pulmonary regurgitation with transpulmonary mean and peak gradients of 46 and 76.7 mmHg, respectively, was also reported. Preoperative computed tomography (CT) scan showed evidence for prior craniotomy, acute SAH with blood within third and fourth ventricles, and within the pre-pontine cistern as well (Fisher 3) (9).

An endovascular procedure was planned for definitive treatment. During the night of admission, oral nimodipine was administered which resulted in profound hypotension exacerbated by an impaired cardiovascular autoregulation. Therefore, fluid administration and a norepinephrine infusion were started. Nimodipine was stopped and the patient was gradually weaned off vasopressors.

On the day of procedure, the radial artery was cannulated to monitor the BP. The preoperative vital signs remained stable with BP = 94/52 mmHg, HR = 76/min with sinus rhythm, and SpO_2_ = 86% (room air). After preoxygenation, anesthesia was induced with IV fentanyl 100 mcg, etomidate 20 mg, and rocuronium 30 mg. The patient was intubated with a size 6 endotracheal tube using a McGrath video laryngoscope. Desflurane 2.5–4.3% was used for anesthesia maintenance, supplemented with muscle relaxation with vecuronium. Monitoring included standard anesthesia monitors, bispectral index, twitch stimulator, invasive central venous pressure (CVP), and arterial pressure monitoring. A cardiologist, cardioanesthesiologist, and a neuroanesthesiologist were in the operating room and available during the procedure. Intraoperative transesophageal echocardiography (TEE) was considered and made available at the bedside, although it was not utilized due to its interference with the cranial fluoroscopy imaging. Mechanical ventilation using pressure-controlled ventilation mode was used throughout the case with the following parameters: peak airway pressure 15 cmH_2_O, FiO_2_ 0.4–0.6, and fresh gas flow 1–1.5 L/min. Normocapnia was maintained throughout the surgery. CVP remained stable at 16–22 mmHg.

A right external ventricular drain was inserted at Kocher’s point obtaining spontaneous egress of cerebrospinal fluid (CSF). The drain was tunneled and fixed in place with no associated complications. In a second surgical timeout, a diagnostic cerebral arteriography was performed through the left vertebral artery with previous right common femoral artery catheterization. Antero–posterior, lateral, and oblique images of the intracranial circulation were performed at this point. Likewise, other images including 3D rotational spinning angiography were obtained from the right and left internal carotid arteries.

The posterior communicating artery aneurysm was successfully catheterized using a Synchro2^®^ microwire through the left-middle cerebral artery. Embolization of the aneurysm was achieved by using a MicroVention MicroPlex-10 4 mm x 8 cm HyperSoft 3D coil followed by one MicoPlex-10 MicroVention 3.5 mm x 5 cm HyperSoft 3D, a MicroPlex-10 MicroVention 3.5 mm x 5 cm HyperSoft 3D, a MicroPlex-10 MicroVention 3 mm x 6 cm HyperSoft 3D, and a MicroPlex-10, 2.5 mm x 6 cm coil respectively. Another series of images were obtained after coiling of the aneurysm and the catheter was withdrawn from the body applying manual pressure in order to obtain hemostasis.

The estimated blood loss during the procedure was minimal (less than 50 mL), and the total intraoperative fluid intake was 500 mL of crystalloids. The patient remained hemodynamically stable during the surgical procedure and did not require any vasopressor infusion.

**Figure 2:**
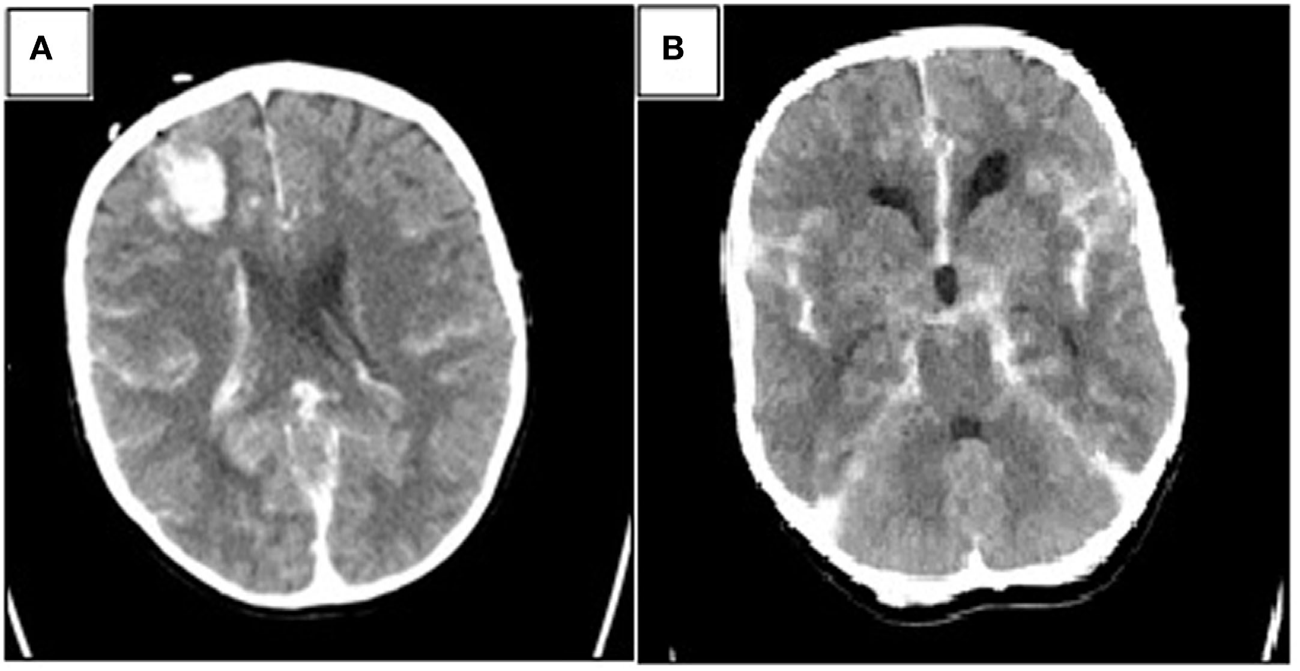
Postoperative head computed tomography (CT) showing **(A)** small amount of hemorrhage along the choroid plexus with a small hematoma in the frontal right lobe corresponding with the ventricular shunt tap and **(B)** basal cisterns with hemorrhage (Fisher 3).

The total length of the procedure was 72 min. At the end of the surgery, neuromuscular blockade was reversed and standard antiemetic prophylaxis was administered. After regaining consciousness, the patient was uneventfully extubated and transferred to the intensive care unit. Postoperative CT scan showed a small hematoma in the right frontal lobe (corresponding with the ventricular shunt tap), SAH, intraventricular and basal cisterns hemorrhage, and hydrocephalus (Fisher 3—Figures 2A,B).

The postoperative course was unremarkable. The patient was discharged home on POD 17 with home health support established.

## Discussion

Intracranial bleeding is a major cause of mortality in patients with ALGS (10). Paradoxically, cerebrovascular abnormalities such as aneurysms and vascular stenosis have been reported in 38% of asymptomatic patients with ALGS who underwent investigational magnetic resonance imaging (MRI). Therefore, MRI should be considered an important tool in identifying potentially treatable intracranial vascular abnormalities, to avoid fatal complications such as infarcts or intracranial bleeding (11).

Management of cerebral aneurysmal rupture and concomitant SAH is even more challenging in patients with congenital cardiac disease. Cerebral vasospasm and aneurysmal rebleeding are both life-threatening complications that significantly increase mortality and worsen neurological outcomes (12). Cerebral vasospasm frequently appears between the third and ninth days after an SAH episode (13, 14). The underlying pathophysiological mechanisms of vasospasm and rebleeding may include increased intracranial pressure, cerebral herniation, development of acute occlusive hydrocephalus, and stroke (15, 16). In our patient, preoperative head CT reported hydrocephalus, although neither aneurysmal rebleeding nor vasospasm occurred during her hospital stay.

Harrod et al. published an extensive review dedicated to pathophysiologic responses after an aneurysm rupture analyzing several variables associated with vasospasm after SAH due to ruptured aneurysms. A large volume of blood with presence of clots had a strong correlation with the onset of vasospasm (13). Prophylactic therapies are commonly used in order to reduce the risk of vasospasm. Triple “H” therapy (hypervolemia, hypertension, and hemodilution) has been associated with enhanced cerebral perfusion and reduced risk of stroke (level B of evidence) (17). Nevertheless, many studies have shown that triple H therapy does not improve patients’ outcomes and can even be detrimental (18). The American Heart Association and American Society of Anesthesiologists 2012 guidelines recommend maintaining a normovolemic state in order to prevent delayed cerebral ischemia (Class I, level of evidence B). Moreover, induced hypervolemia is no longer recommended to prevent the onset of vasospasms (Class III, level of evidence B) (12).

Calcium channel blockers such as verapamil and nimodipine are commonly used in the prevention and treatment of vasospasm after SAH (19). Despite existing controversies in vasospasm prophylaxis therapy after aneurysmal SAH, better neurologic outcomes have been shown with oral or intravenous nimodipine when compared with placebo (12, 20, 21). However, nimodipine may decrease venous return, BP, and systemic vascular resistance (SVR) and its administration in patients with complex congenital cardiovascular disease should be strictly monitored. Recently, intravenous sildenafil has been also used in phase-I safety studies for cerebral vasospasm after SAH (22).

### Intraoperative Considerations

Neurosurgical clipping and endovascular coiling are widely known surgical treatment options for ruptured cerebrovascular aneurysms. Extensive literature regarding both techniques has been published comparing short- and long-term outcomes such as neurological outcomes, quality of life, and procedure-related complications (23, 24).

Greater fluid shift and intraoperative bleeding associated with open neurosurgery would have increased the risk of fluid overload and intraoperative heart failure in our patient. Additionally, considerable blood loss and concomitant hypotension may require the use of intraoperative hemodynamic support such as systemic sympathomimetic drugs (e.g., norepinephrine), which in the setting of high pulmonary BP may worsen right ventricle performance. Pulmonary vasoconstriction, increased pulmonary afterload, and decreased oxygen saturation are some of the ancillary effects of norepinephrine use (25, 26).

Our patient had previously undergone an RVOT patch augmentation with improvement of the fixed component of her shunt (right ventricular obstruction). Nevertheless, she was very sensitive to changes in the pulmonary vascular resistance (PVR)/SVR balance as observed during nimodipine administration. Undoubtedly, the RVOT patch augmentation should have decreased the right ventricle afterload by correcting the proximal stenosis in her cardiac anomaly. However, her bilateral branch PAS remained uncorrected, causing a fixed pulmonary hypertensive state with a diminished response to pulmonary vasodilators. Thus, the increased right ventricular size described during the preoperative echocardiogram in our patient is the result of an adaptive process of the right ventricle in order to increase preload. Probably, this adaptation has been taking place since birth, involving several morphological and functional changes known as ventricular remodeling (27).

Intraoperative management of TOF patients should focus on decreasing R–L shunt. This shunt reduction (28) could be achieved by:

(a) Increasing blood flow in the pulmonary artery.
(b) Maintaining systemic BP at acceptable values.
(c) Using vasopressors as needed in order to maintain SVR.
(d) Increasing RV unloading by reducing PVR.
(e) Avoiding systemic hypotension and tachycardia.

Since transesophageal echocardiography was available but not performed due to the aforementioned reasons, CVP, invasive arterial BP, and SpO_2_ monitoring were used as alternative variables in order to evaluate and to identify intraoperative shunt direction in our patient. These hemodynamic variables were closely monitored in order to avoid significant changes such as severe hypertension with subsequent increased risk of bleeding and cerebral ischemia (29). Somatosensory evoked potentials monitoring has been also used to detect cerebral ischemia and guide BP management (30).

Severity of the right ventricular obstruction and the effect of anesthetic drugs on SVR and PVR should be considered in order to determine the anesthetic management for these patients. The choice of anesthetics with reduced cardiovascular effects, low hepatic metabolism, and faster clearance was paramount in our patient.

Induction time with inhalational agents is prolonged in patients with R–L shunts, and therefore intravenous induction is preferable (28, 31). However, Yu et al. showed that sedation with propofol increases preload dependency in critically ill patients probably due to the attenuation of the autonomic responses and potential decrease in peripheral resistance (32). Sympathomimetic effect and cardiac stability associated with etomidate make it the drug of choice for anesthesia induction in patients with cardiovascular impairment (33).

Desflurane is widely used for maintenance of general anesthesia in neurosurgical pediatric patients. Ghoneim et al. reported a faster emergence from anesthesia with the administration of desflurane or sevoflurane found in 60 patients after supratentorial tumor resection when compared with isoflurane. In addition, significant differences were found when comparing the time to achieve an Aldrete score ≥9 between desflurane or sevoflurane and isoflurane. More than 50% of the patients in the desflurane and sevoflurane groups were extubated within 15 min after discontinuation of the agent whereas no patients in the isoflurane group were extubated within this range (34).

## Conclusion

In this case report, we described a successful perioperative management of a 17-year-old female patient with ALGS, unrepaired TOF, open RVOT, and PAS who presented to the emergency department with SAH. The achievement of perioperative goals in our patient relied on a multidisciplinary approach throughout the entire process. Clinical decisions were made by surgeons in conjunction with a cardiologist, a cardioanesthesiologist, and a neuroanesthesiologist who were all present in the operating room. Therefore, we consider that a multidisciplinary intervention in specialized centers offers a better alternative for the perioperative management of patients with specific comorbidities where neurologic and cardiovascular dysfunction overlaps.

## Ethics Statement

Informed consent: patient and her guardian signed consent allowing us to present this case report using her medical information. All data has been de-identified and informed consent has been filed in accordance with the statement below: The Ohio State University, College of Medicine, and Office of Research. Case studies do not require IRB approval, so what authorizations are needed from patients to publish a case study? The Release of Information (ROI) form should be used to get the patient’s authorization for their de-identified information to be used in a case study. The case study must be de-identified. The form should say that they authorize the staff writing the case study to release information to x journal/meeting for the purpose of a de-identified case study. Need more information? https://medicine.osu.edu/research/clinical_research/faqs/pages/case-studies.aspx.

## Author Contributions

All authors contributed equally to the development of this manuscript.

## Conflict of Interest Statement

The authors declare that the research was conducted in the absence of any commercial or financial relationships that could be construed as a potential conflict of interest. The reviewer CB and handling editor declared their shared affiliation.
